# Woman’s Medical College

**Published:** 1881-03

**Authors:** 


### ARTICLE XII.

### WOMAN'S MEDICAL COLLEGE.

The Eleventh Annual Commencement of this institution was held in Centenary Church, March 1. There were seventeen graduates, one of whom did not receive her diploma immediately, because she had not yet attained her twenty-first year. Number of students in attendance through the year, seventy-five. Conferring of degrees by Pres. Wm. H. Byford, M.D., S. H. Stephenson, M.D. The only female professor present distributed the boquets in a very elegant manner.

The class valedictory was rendered by Dr. Ella M. Gilchrist, who gave a brief history of the practice of medicine by women since the beginning of the world, stating that long before the Christian era, women's medical colleges had existed in Egypt. She alluded to the achievements of Russian female doctors in the last Turco-Russian war, and ended this sketch in stating that Elizabeth Blackwell was the first woman who received a medical diploma in this country (1849). Massachusetts has founded the first medical school for woman in modern times. She then enumerated the duties which her class had just entered upon and bid farewell to the faculty. All persons present enjoyed the address to the utmost, and repeatedly applauded the speaker.

The faculty address was given by Prof. Wm. J. Maynard, M.D., and chiefly consisted in timely advice, and the good wishes of the faculty.

The present is the largest class that ever graduated from the college. H. D. V.

